# The effects of nudging and pricing on healthy food purchasing behavior in a virtual supermarket setting: a randomized experiment

**DOI:** 10.1186/s12966-020-01005-7

**Published:** 2020-08-03

**Authors:** Jody C. Hoenink, Joreintje D. Mackenbach, Wilma Waterlander, Jeroen Lakerveld, Nynke van der Laan, Joline W. J. Beulens

**Affiliations:** 1 Amsterdam UMC, Vrije Universiteit Amsterdam, Department of Epidemiology and Data Science, Amsterdam Public Health research institute, De Boelelaan, 1117 Amsterdam, the Netherlands; 2 Upstream Team, www.upstreamteam.nl, Amsterdam UMC, De Boelelaan 1089a, 1081 HV Amsterdam, the Netherlands; 3 Department of Public Health, Amsterdam UMC, University of Amsterdam, Amsterdam Public Health Research Institute, Meibergdreef 9, Amsterdam, the Netherlands; 4 Julius Center for Health Sciences and Primary Care, University Medical Center Utrecht, Utrecht University, Utrecht, the Netherlands; 5 Department of Communication and Cognition, Tilburg University, Tilburg, the Netherlands

**Keywords:** Socio-economic inequalities, Food purchases, Taxes, Subsidies, Price decreases, Randomized trial, Intervention study, Policy intervention

## Abstract

**Background:**

Evidence on what strategies - or combination of strategies - are most effective and equitable in promoting healthier diets is needed. This study examined the efficacy of nudging and pricing strategies on increasing healthy food purchases and the potential differential effect by socio-economic position (SEP) among Dutch adults in a virtual supermarket.

**Methods:**

A randomized study design was conducted within a virtual supermarket (SN VirtuMart). Participants were exposed to five within-subject study conditions (control, nudging, pricing, price salience and price salience with nudging) and randomized to one of three between-subject study arms (a 25% price increase on unhealthy products, a 25% discount on healthy products, or a 25% price increase and discount). In total, 455 participants of low and high SEP (using either education or income as proxy) were randomized to conduct their weekly shopping in a virtual supermarket for five consecutive weeks. The primary outcome included the percentage of healthy purchases. Data were analyzed using linear mixed models.

**Results:**

In total, 346 (76%) adults completed all five shops within the SN VirtuMart. Median age was 32.5, 49.2% had high education and 32.8% had high income. Out of the 12 conditions, four conditions were statistically significantly different from the control condition. Nudging and non-salient pricing strategies alone did not statistically significantly increase healthy food purchases, whereas a combination of salient price increases and discounts led to an increase in the percentage of healthy food purchases (B 4.5, 95%CI 2.6; 6.4). Combining salient pricing and nudging strategies led to increases in the percentage of healthy products in all three pricing arms, with largest effects found in the combined price increase and discount arm (B = 4.0, 95%CI = 2.0; 6.0). Effects were not modified by SEP.

**Conclusions:**

Combining health-related price increases and discounts and combining these salient pricing strategies with nudges in a supermarket setting seems to stimulate healthy food purchases for both low and high SEP populations. However, further research in real-world settings is needed.

**Trial registration:**

This randomized trial (NTR7293) was registered in the Dutch trial registry (www.trialregister.nl).

## Background

Population diets have shifted towards a greater consumption of unhealthy foods [1, 2], leading to an increase in the prevalence of diet-related chronic diseases such as type 2 diabetes [3]. Interventions in food purchasing settings (e.g., supermarkets) are promising for the prevention of these diet-related chronic diseases. In particular, food taxes and subsidies aimed at improving dietary intake are becoming increasingly popular and the evidence base for the effectiveness of these strategies is rapidly growing [4–6]. A systematic review investigating the prospective impact of pricing strategies on dietary consumption generally found that these are effective in increasing healthy food intake and decreasing unhealthy food intake [7]. Combining pricing strategies with communication about the price changes (i.e., salience) may further enhance their effectiveness [8]. Furthermore, even though evidence indicates that discounts on healthy foods significantly increase the purchases of the targeted foods [5], theory [8] and evidence [9] suggests that consumers respond more strongly to price increases, as they are experienced by consumers as losses, than to price discounts, which are perceived as gains.

Alternatively, nudges are often seen as a less invasive way of steering consumers towards healthier behaviors. A nudge can be defined as any aspect of the choice architecture that alter people’s behavior in a predictable way without forbidding any options or significantly changing their economic incentives [10]. The use of nudges to promote healthier diets has been shown to be acceptable to the public [11], feasible in a range of settings [12] and, depending on context and type of nudge, moderately effective [13]. Salience nudges (i.e., drawing an individual’s attention towards a particular option) targeted at healthy products may be especially promising for supermarket environments, as they target the same type of decision making as traditional marketing strategies. However, to what extent salience nudges are effective in promoting healthier purchases in supermarkets is yet to be determined.

Due to budgetary constraints, individuals with low SEP may react more strongly to (salient) price changes compared to individuals with high SEP [9]. There are mixed findings regarding the differential effects of economic interventions on food purchases by SEP, with one study finding more healthy purchases in middle compared to low SEP women [14] and other studies finding no difference in healthy purchases by low compared to high SEP adults [15, 16]. Given the mixed results found for pricing strategies, more research is needed regarding the differential effects of SEP in the association between pricing strategies and food purchasing behavior. No studies to date have investigated the differential effects of salient nudging strategies across levels of SEP in a food retail setting. Identifying which nudging and/or pricing strategies are effective and equitable is important for wider implementation of such strategies.

While the independent effects of non-salient pricing and nudging strategies have been evaluated in previous studies [5, 13], combining both strategies could lead to larger health gains. There is a need for experimental studies in a supermarket environment that look into (1) the combined effects of nudges and pricing strategies, (2) the differential effects of health-related price increases and discounts, (3) the added value of salient price increases and discounts compared to non-salient price changes, and (4) whether effects differ between low and high SEP populations. Therefore, this study aims to examine the efficacy of nudging and several pricing strategies on increasing healthy food purchasing behavior (by increasing healthy food purchases and decreasing unhealthy food purchases) and effect modification by SEP among Dutch adults in a virtual supermarket setting. We hypothesize that combining all nudging and pricing strategies will be most effective and that nudging strategies alone will be least effective in increasing healthy food purchases. Furthermore, we expect lower SEP participants to react more strongly to the pricing strategies compared to higher SEP participants. A virtual supermarket was used to answer these research questions because this offers a practical and affordable means to test the efficacy of nudging and pricing strategies before these are implemented in real-world settings [17]. Virtual supermarkets are a valid tool to investigate the effect of pricing strategies on food purchases [18] and the purchases conducted within virtual supermarkets resemble those made in real life [19, 20].

## Methods

We used a three-dimensional (3D) web-based virtual supermarket (the SN VirtuMart) to test the efficacy of nudging, pricing and combined nudging and pricing strategies. This experimental study was part of the ‘Sustainable Prevention of Cardiometabolic Risk through Nudging Health Behaviors’ (Supreme Nudge) project [21]. This randomized trial (NTR7293) was registered in the Dutch trial registry.

The virtual supermarket was designed to simulate a real-life shopping experience by imitating a typical Dutch supermarket (i.e., layout of the shelves, colors, products and product prices). The virtual supermarket included almost 1200 unique name-brand and budget-brand products categorized into 12 food groups. The quantity and variety of products was such that participants with a variety of household sizes and budgets were able to do their weekly shopping in the virtual supermarket. For example, the proportion of healthy products within the SN VirtuMart was comparable to the proportion found in real-life supermarkets. In the SN VirtuMart, 19% (221 out of 1175) of products were considered to be healthy (as based on the Dutch dietary guidelines [22]) compared to 16% of products in Dutch supermarkets that are healthy in terms of being fresh, unprocessed or lightly processed foods [23]. Common marketing, branding and promotion techniques as well as sounds and background noise were used in all shopping conditions to simulate real-life supermarket shopping experiences. The virtual supermarket software was pilot-tested prior to the study, but no formal usability test was conducted. The virtual supermarket was designed by co-author L.N. van der Laan (Van der Laan LN, Papies EK, Ly A, Smeets PA: How health goal priming promotes healthy food choice: a virtual reality fMRI study, submitted). The study design and procedures were approved by the Medical Ethics Review Committee of VU University Medical Centre (OHRP: IRB00002911). More information regarding the SN VirtuMart program and selection of foods and beverages can be found in the Additional Material.

### Study design

This study used a mixed randomized experimental study design consisting of five study conditions (within-subject design) and three study arms (between-subject design). Participants were randomized into one of the three study arms (25% price increases, 25% price discounts, or 25% price increases and discounts) and within these arms exposed to five study conditions (control, nudging, pricing, price salience and price salience with nudging). A 25% price change was chosen based on the finding that at least a 20% price change is needed to result in significant effects on population health [24] and based on discussions with a Dutch supermarket chain regarding what price changes would be feasible in real-world supermarkets [25]. The order in which the participants received the five study conditions was also randomized. Table 1 displays the study design.

**Table 1:** Study design

Condition^a^	Arm 1 – Price increases	Arm 2 – Price discounts	Arm 3 – Price increases and discounts
Control condition	Control	Control	Control
Nudging condition	Nudging	Nudging	Nudging
Pricing condition	Price increases	Price discounts	Price increases and discounts
Price salience condition	Salient price increases	Salient price discounts	Salient price increases and discounts
Price salience with nudge condition	Nudging + salient price increases	Nudging + salient price discounts	Nudging + salient price increases and discounts

^a^The order in which participants received the conditions was randomized

The control condition represented regular supermarket price promotions and product placement. The nudge condition included salience nudges to promote high-fiber products, frozen vegetables and low-fat dairy products. Salience nudges are nudges that draw individual’s attention towards a particular option through for example the use of arrows or frames (Additional Material). The price condition represented discounts in the prices of healthy products and/or increases in the prices of unhealthy products, depending on study arm. In the price salience condition, price increases and discounts were implemented and communicated to participants. Price increases were communicated to participants by showing a newspaper announcement of an unhealthy food tax of 25% on their screen, before they entered the supermarket. Price discounts were communicated with price promotion signs within the virtual supermarket. The last shopping condition was a combination of the nudge and price salience condition.

Participants randomized to the price increases arm were exposed to 25% price increases on 37% of the available unhealthy products (*n* = 356/955) (i.e., pizza, white bread, confectionary, sugary drinks, high-fat and/or high-sugar dairy products, salted nuts and sweet bread spreads). Participants randomized to the price discounts arm were exposed to 25% discounts on 89% of healthy products (*n* = 195/220) available within the virtual supermarket (i.e., all fresh and frozen fruits and vegetables, canned vegetables, high-fiber bread and bread alternatives, whole-wheat pasta and rice, low-fat and low-sugar dairy products, fish, unsalted nuts, water and tea). Participants randomized to the price increases and discounts arm were exposed to both the price increases and decreases. Products were considered to be healthy based on the Dutch dietary guidelines [22] and unhealthy products were all products not recommended by these guidelines.

### Randomization and masking

Two online block randomizer generators were used to allocate participants equally to the arms and to determine the order of the conditions. Participants were masked to the nature of the conditions they were assigned to and were not aware that the study aimed to evaluate the effect of nudging and pricing strategies. Instead, they were told the study was about shopping behaviors in general. The online registration, randomization procedure and analysis of the data were all conducted by the research team.

### Participants and recruitment

Details on the recruitment of participants are described elsewhere [17]. Briefly, the aim was to include an approximately equal distribution of low and high SEP individuals. Participants were recruited from the general Dutch population through targeted Facebook advertisements and home-delivered flyers in selected low-SEP neighborhoods throughout the Netherlands. The advertisements and flyers directed participants to the registration website. Upon signing online informed consent, participants received a questionnaire assessing eligibility criteria and socio-demographic characteristics. Inclusion criteria were: being an adult (18 years or older); being the main household shopper; being able to read in Dutch; and to have an email address. Participants were excluded if another household member was already participating in the study, or if they did not have a computer or laptop. Participants who met the inclusion criteria and completed a training task of ‘buying’ five specific products were included in the study.

### Sample size

The sample size calculation was based on previous literature [12, 26]. We determined that a sample of 150 participants (i.e., 50 participants in each pricing arm) completing all five conditions would be adequate to detect, among others, a target difference of 135 (SD: 370) grams of vegetables per week between the control condition and nudging condition (level of significance 0.05, power > 0.90). Larger differences were expected for the pricing conditions compared to the nudging condition. Also, we expected the salient pricing strategies to be larger than the non-salient pricing strategies and that the combination of price increases and discounts would be larger than the single pricing strategies. Lastly, the largest difference was expected when combining all strategies (i.e., nudges and pricing strategies and price increases and discounts). The same standard deviation of 370 was used for all conditions. In order to be able to have enough power to stratify the results by low and high SEP, we aimed to include double the sample, leading to a total sample of 300 participants who completed all five shops. We aimed to oversample participants and monitor their drop-out so that we would end up with 300 participants. More information regarding the sample size calculation has been reported elsewhere [17].

### Purchasing task

Participants were asked to perform five shops in the virtual supermarket over five consecutive weeks. During each virtual supermarket visit, participants were asked to do their regular weekly household groceries, using a virtual budget. Participants’ shopping budgets (eight categories) were based on self-reported actual grocery shopping budgets and they were only able to leave the supermarket at the check-out if they had spent between 50 and 125% of their budget [17].

### Outcome measures

The primary outcome was the percentage of healthy products based on the healthy and unhealthy purchases in grams per week. This outcome measure was chosen as pricing and nudging strategies aim to increase the proportion of healthy purchases through increasing healthy purchases and/or decreasing unhealthy purchases. The total grams of healthy and unhealthy products purchased per week were used as secondary outcome measures. These secondary outcome measures can help explain whether the percentage of healthy purchases changed due to; 1) a change in healthy purchases (which is expected in the cases of price discounts and nudges), 2) a change in unhealthy purchases (which is expected in the case of price increases), 3) or a change in both healthy and unhealthy purchases (which is expected when combining price increases and discounts). In the original trial registry, the primary outcomes included the percentage of healthy purchases as well as the purchases of healthy and unhealthy foods. However, given that price increases mostly affect the purchases of unhealthy foods and price discounts the purchases of healthy foods, it would not be very informative to calculate the purchases of healthy and unhealthy foods for the overall sample. These were therefore classified as secondary outcome measures.

To investigate if participants spent more money in the virtual supermarket, especially when exposed to price increases, we also investigated the total amount spent in the virtual supermarket per week in Euros (tertiary outcome). Sensitivity analyses were conducted with the number of healthy and unhealthy products purchased per week and the percentage healthy products based on the number of healthy and unhealthy products purchased.

### Covariates

The baseline questionnaire asked participants for their age, sex, educational attainment, household net monthly income, usual weekly budget spent on groceries, weight and height. Self-reported height and weight were used to calculate body mass index (BMI - kg/m^2^). BMI was dichotomized according at overweight status (BMI ≥ 25 kg/m^2^).

Educational level and income were used as two separate proxies for SEP because they assess different aspects of SEP. Educational level was categorized into two groups: low educational level included those who completed primary education, intermediate vocational education and higher secondary education, and high educational level included those who completed higher vocational education or university. In order to adjust household income for household size, we used the OECD-modified equivalence scale [27]. After this adjustment, low income was defined as a value equal to or below the median individual income of €1743 per month and high income was defined as all values above this median individual income. The average monthly gross income in the Netherlands in 2017 was €2667 [28].

### Statistical analyses

Descriptive statistics for socio-demographic variables and the outcome variables were reported using percentages, means and standard deviations or medians and interquartile distances in case of non-normality. Mean changes from the control condition were analyzed using a maximum likelihood-based repeated measures approach including a random intercept for participants to account for the clustering of shops within participants. An exchangeable covariance structure was used to model the within-participant errors. The research aim regarding the independent and combined effect of nudging and several pricing strategies was assessed using a linear mixed model with the percentage of healthy purchases as the only outcome (aim 1). Only for the single nudging condition, a linear mixed model was used to investigate the effect of nudges on the secondary outcome measures (i.e., healthy and unhealthy purchases) without stratifying for the pricing arms. The differential effects of price increases and discounts (aim 2) was assessed by stratifying the linear mixed model by the three pricing arms for both the primary (percentage of healthy purchases) and secondary outcome measures (amount of healthy and unhealthy purchases in grams). In order to investigate the third aim regarding the added value of salient price increases, the reference group included the pricing only condition instead of the control condition. To investigate effect modification by SEP (aim 4), the analyses were stratified for SEP indicators (i.e. educational level and income separately). For the within-subject analyses, participants that completed at least two shops contributed to the analyses. Whether there were statistical differences between SEP strata was examined by comparing the model without interactions with the model with interactions between conditions and SEP using the likelihood ratio test, separately for the three pricing arms. Additionally, the total amount spent in the virtual supermarket for each experimental condition compared to the control condition was analyzed and can be found in the additional material. Furthermore, sex differences were investigated because males and females may react differently to price changes due to differences in competing factors such as perceived quality, price, taste and habit [29] (Additional Material). Lastly, in order to determine whether a possible order or learning effect occurred due to the study design, we investigated the effect of the intervention period (ranging from week 1 to week 5) on the percentage of healthy purchases. Also, we compared the unadjusted beta coefficients to the period adjusted beta coefficients of the effect of the experimental conditions on the percentage of healthy purchases (Additional Material).

Analyses were conducted in STATA version 14.1 and the absence of zero in the 95% confidence interval or a *p*-value of 0.05 or smaller was regarded as a statistically significant effect. We did not adjust for multiple testing given the fact that we used a single primary outcome, and the findings from the secondary outcomes were used to explain the primary outcome findings.

## Results

**Fig. 1:**
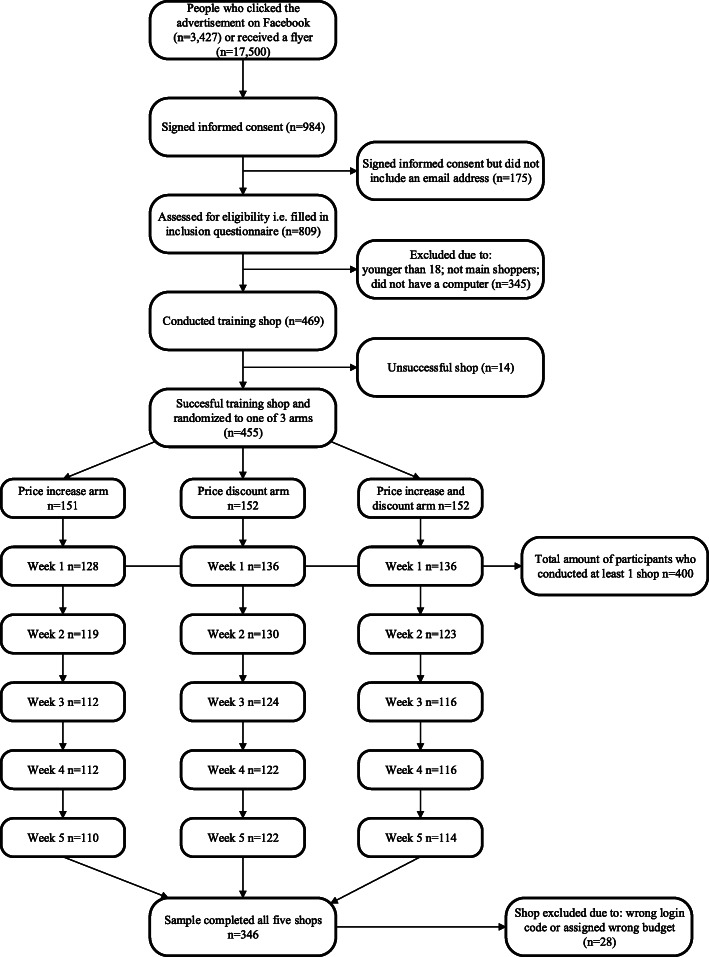
Flow chart of the selection of participants’ process

In the winter of 2018/2019, 455 participants were randomized to one of three arms, 400 (88%) participants conducted at least one shop, 346 (76%) participants completed all five shops and 318 (70%) participants had usable data for all five shops (e.g., data where log in codes with the wrong budget assigned were excluded) (Fig. 1). The median age of participants was 30.0 (IQD 24.0) and more than 60% were female, which varied from 59 to 65% depending on the study arm (Table 2). Mean BMI of participants at baseline was 24.7 (SD 4.8), and this was fairly equal across the three arms. A little over 49% of participants had a high educational level and almost 33% had a high income. In the control condition, approximately 46% of participants’ purchases consisted of healthy foods, and participants on average spent 100.2 Euros (SD 44.4) per week on their groceries (Additional Table 1).

**Table 2:** Socio-demographic characteristics of the study population

Sociodemographic characteristics	25% price increase arm (*N* = 128)	25% price discount arm (*N* = 136)	25% price increase and discount arm (*N* = 136)	Total sample (*N* = 400)
Median age in years (IQR)	32.5 (25.0)	39.5 (24.5)	30.0 (23.0)	30.0 (24.0)
Sex (% female)	59.4%	58.8%	65.4%	61.2%
Mean BMI (SD)^a^	24.6 (4.8)	24.6 (4.9)	25.1 (4.7)	24.7 (4.8)
Overweight status (% overweight or obese)^a^	34.9%	35.8%	41.2%	37.3%
Educational level (% highly educated)	49.2%	42.7%	44.1%	45.3%
Income (% high income)^b,c^	40.9%	26.1%	31.9%	32.8%
Employment situation				
*% Full time job*	29.7%	22.8%	22.8%	25.0%
*% Part time job*	25.8%	25.0%	25.7%	25.6%
*% Student*	20.3%	26.5%	29.4%	25.5%
*% Unemployed*^d^	22.7%	22.8%	17.7%	21.0%
*% Entrepreneur or other*	2.4%	3.0%	4.4%	2.3%
Household composition				
*Mean number of adults in the household*	1.9 (0.8)	1.9 (1.1)	1.9 (0.8)	1.9 (0.9)
*Mean number of children in the household*	0.5 (0.9)	0.5 (0.9)	0.5 (1.0)	0.5 (0.9)

*Abbreviations*: *IQR* Interquartile range, *SD* Standard Deviation

^a^9 Missing values

^b^4 missing values

^c^Calculated using the OECD-modified scale and the median cut-off value was €1743

^d^Includes those who are retired, unemployed, unable to work and/or receiving social benefits, and housewives/men

### The independent and combined effects of nudging and pricing strategies

In Table 3, the effects of the experimental conditions on the percentage of healthy purchases for the total sample are displayed. The nudging only (B 0.5, 95%CI -0.6; 1.6) and pricing only (B 0.4, 95%CI -0.7; 1.6) conditions did not increase the *percentage of healthy purchases* as compared to the control condition. The price salience and combined price salience and nudging conditions led to a 2.6% (95%CI 1.4; 3.7) and 3.1% (95%CI 1.9; 4.3) increase in the *percentage of healthy products*, respectively.

**Table 3:** Effects of nudging and pricing strategies on the outcome measures for the total sample^a^ and stratified by pricing arms

Conditions	25% increase arm (*n* = 128)		25% discount arm (*n* = 136)		25% increase and discount arm (*n* = 136)		Total sample (*n* = 400)	
	B	95%CI	B	95%CI	B	95%CI	B	95%CI
Percentage of healthy purchases (primary outcome)								
Control	Ref.		Ref.		Ref.		Ref.	
Nudging	−0.5	−2.5; 1.5	0.2	−1.8; 2.2	1.8	−0.1; 3.7	0.5	−0.6; 1.6
Pricing	0.0	−1.9; 2.0	0.5	−1.5; 2.5	0.7	−1.2; 2.6	0.4	−0.7; 1.6
Price salience	1.4	−0.6; 3.4	1.9	−0.1; 3.9	**4.5**^b^	**2.6; 6.4**	**2.6**^a^	**1.4; 3.7**
Price salience and nudging	**3.0**	**1.1; 5.0**	**2.3**	**0.3; 4.3**	**4.0**	**2.0; 6.0**	**3.1**	**1.9; 4.3**
Healthy purchases in grams (secondary outcome)								
Control	Ref.		Ref.		Ref.		NA	
Nudging	− 416.3	− 1173.7; 341.2	− 286.4	− 1050.4; 477.5	68.4	− 741.1; 877.9	NA	NA
Pricing	− 695.5	− 1451.2; 60.1	44.2	− 720.7; 809.0	−125.8	− 949.5; 697.8	NA	NA
Price salience	−29.0	− 791.2; 733.2	**1038.5**^b^	**274.6; 1802.3**	**1025.7**^b^	**180.8; 1870.5**	NA	NA
Price salience and nudging	−41.1	− 804.1; 721.9	440.6	− 320.3; 1201.5	**1646.3**	**734.7; 2557.8**	NA	NA
Unhealthy purchases in grams (secondary outcome)								
Control	Ref.		Ref.		Ref.		NA	
Nudging	− 534.6	− 1480.6; 411.5	− 756.4	− 1781.4; 271.9	− 907.4	− 1868.4; 53.6	NA	NA
Pricing	**− 1194.0**	**− 2134.6; − 253.3**	−534.7	− 1569.2; 499.9	− 608.8	− 1576.6; 358.9	NA	NA
Price salience	**− 991.5**	**− 1935.6; −47.3**	90.0	− 941.9; 1122.0	**− 1680.8**^b^	**− 2657.0; − 704.5**	NA	NA
Price salience and nudging	**−1802.7**	**− 2742.3; − 863.2**	− 993.0	− 2028.8; 32.9	− 885.5	− 1917.1; 146.0	NA	NA

Bold values are statistically significant

*Abbreviations*: *B* Beta regression coefficient, *CI* Confidence interval, *NA* Not Applicable, *Ref* Reference group

^a^Only applicable for the primary outcome measure percentage of healthy purchases

^b^Price salience condition statistically significantly differs from pricing condition

The effect of the nudges on the amount of healthy and unhealthy purchases (secondary outcomes) was investigated without stratifying the results by pricing arm. Nudging only did not statistically significantly increase *healthy purchases* (B -209.2, 95%CI -658.0; 239.5), but did statistically significantly decrease *unhealthy purchases* by 737.7 g a week per household (95%CI -1306.2; − 169.3).

### Efficacy of nudging and several pricing strategies according to study arm

Table 3 also presents the effects of the experimental conditions compared to the control condition stratified by study arm (i.e., separate for the three different pricing arms) for the primary and secondary outcome measures. Regarding the outcome *percentage of healthy purchases*, neither the single price increase arm nor the single price discount arm resulted in statistically significant differences compared to the control condition. Combined salient price increases and discounts statistically significantly increased the *percentage of healthy purchases* with 4.5% (95%CI 2.6; 6.4). Within all three study arms, combining nudging and pricing strategies led to a statistically significant increase in the *percentage of healthy purchases*, with the largest increase found in the combined price increase and discount arm (B 4.0, 95%CI 2.0;6.0).

Mixed results were found for the secondary outcome measures *healthy purchases* and *unhealthy purchases*. A salient 25% price discount and both salient price increases and discounts statistically significantly increased *healthy purchases* by 1600 g compared to the control condition and combining the salient price increases and discounts with nudging led to the highest increase of *healthy purchases* (B 1646.3, 95%CI 734.7; 2557.8). Statistically significant decreases were found for *unhealthy purchases* in the price only, price salience and combined price salience with nudging conditions, but only for the price increases arm. A combination of salient price increases and discounts also led to a statistically significant decrease in *unhealthy purchases* of 1681.3 g (95%CI -2662.0; − 700.7). No other statistically significant effects were found in either outcome measures (Table 3).

### Added value of making price changes salient

Statistically significant differences between the pricing and price salience conditions can also be seen in Table 3. In almost all cases, if the price salience condition differed statistically significantly from the control condition, it also differed statistically significantly from the pricing condition.

### SEP differences

Table 4 illustrates the effects of independent and combined nudges and pricing strategies for low and high SEP as measured by educational level and income for the percentage of healthy purchases. Overall, no statistically significant differences between low and high SEP for the outcome *percentage of healthy purchases* were found according to the likelihood ratio test (*P* > 0.05). However, some small differences should be noted. A 25% price increase on unhealthy products combined with nudges only had a statistically significant effect on the *percentage of healthy purchases* in low educated participants only. In high educated participants, salient price discounts had a statistically significant effect on the *percentage of healthy purchases* (B 3.2, 95%CI 0.3; 6.2). In the 25% discount arm, stronger effects were found for participants with a low income compared to a high income in the price salience and price salience with nudging conditions. For example, for low income participants in the price salience with nudges condition, the *percentage of healthy purchases* increased by 3**.**5% (95%CI 1**.**2; 5**.**8), while for high income participants no statistically significant increase was found (B -0**.**1, 95%CI -3**.**9; 3**.**7). Stratified results by SEP indicators for the secondary outcome measures can be found in Additional Tables 2 and 3.

**Table 4:** Effects of nudging and pricing strategies on the percentage of healthy purchases stratified by arm and SEP indicators

Conditions	25% increase arm					25% discount arm					25% increase and discount arm				
	Low educational level (*n* = 65)		High educational level (*n* = 63)			Low educational level (*n* = 78)		High educational level (*n* = 58)			Low educational level (*n* = 76)		High educational level (*n* = 60)		
	B	95%CI	B	95%CI	lr-test	B	95%CI	B	95%CI	lr-test	B	95%CI	B	95%CI	lr-test
Control	Ref.		Ref.		0.1	Ref.		Ref.		0.2	Ref.		Ref.		0.3
Nudging	2.0	−0.5; 4.5	−2.7	−5.8; 0.3		−1.3	−3.9; 1.4	2.0	−0.9; 5.0		0.6	−1.9; 3.1	**3.3**	**0.3; 6.2**	
Pricing	2.2	−0.3; 4.6	−2.0	−5.0; 1.1		−0.4	−3.0; 2.3	1.8	−1.2; 4.8		−0.9	−3.3; 1.6	2.7	−0.3; 5.7	
Price salience	1.8	−0.7; 4.3	1.1	−1.9; 4.2		0.9	−1.8; 3.5	**3.2**	**0.3; 6.2**		**2.8**	**0.3; 5.3**	**6.6**	**3.6; 9.6**	
Price salience and nudging	**4.4**	**1.9; 6.8**	1.8	−1.2; 4.9		2.6	−0.0; 5.3	1.9	−1.0; 4.9		**3.5**	**0.8; 6.1**	**4.8**	**1.7; 8.0**	
	Low income level (*n* = 75)		High income level (*n* = 52)			Low income Level (*n* = 99)		High income level (n = 35)			Low income level (*n* = 92)		High income level (*n* = 43)		
	B	95%CI	B	95%CI	lr-test	B	95%CI	B	95%CI	lr-test	B	95%CI	B	95%CI	lr-test
Control	Ref.		Ref.		1.0	Ref.		Ref.		0.2	Ref.		Ref.		0.7
Nudging	−0.5	−3.0; 2.0	− 0.5	− 3.8; 2.8		0.9	−1.3; 3.2	−1.5	−5.4; 2.4		1.6	−0.6;3.9	1.8	−1.6; 5.2	
Pricing	−0.5	−3.0; 2.1	0.8	−2.4; 4.0		0.7	−1.5; 3.0	0.8	−3.2; 4.7		0.9	−1.4; 3.2	−0.6	−4.1; 2.8	
Price salience	1.0	−1.5; 3.6	2.0	−1.3; 5.2		**2.9**	**0.6; 5.2**	−1.3	−5.2; 2.6		**4.0**	**1.7; 6.3**	**5.0**	**1.6; 8.5**	
Price salience and nudging	**2.8**	**0.4; 5.4**	3.1	−0.1; 6.4		**3.5**	**1.2; 5.8**	−0.1	−3.9; 3.7		**4.5**	**2.0; 7.0**	3.0	−0.6; 6.5	

Bold values are statistically significant

*Abbreviations*: *B* Beta regression coefficient, *CI* Confidence interval, *lr-test p*-value of the likelihood-ratio test, *Ref* Reference group

### Additional analyses

The results of the sensitivity analyses with the number of healthy and unhealthy products were similar to the main results (Additional Table 4). The total amount of money spent in the virtual supermarket in the price increases arm did not differ for the experimental conditions compared to the control condition (Additional Table 5). In the discounts arm and the combined price increases and discounts arm, participants decreased the amount spent in the virtual supermarket in several pricing conditions compared to the control condition (Additional Table 5). Overall, no statistically significant differences between males and females were found (Additional Table 6). The intervention period did not influence the percentage of healthy purchases, and the beta regression coefficients barely changed after adjusting the model investigating the effect of the experimental conditions on the percentage healthy purchases for the intervention period (Additional Tables 7 and 8).

## Discussion

In this study we investigated the efficacy of the independent and combined effects of nudging and pricing strategies on food purchasing behavior in a virtual supermarket. Nudges and non-salient price strategies alone had limited effect. Also, salient price increases alone or salient price discounts alone did not increase the percentage of healthy purchases, while the combined salient price increases and discounts increased the percentage of healthy food purchases. Combining these salient price increases and/or discounts with nudges had the strongest effect on the percentage of healthy food purchases. There was limited evidence for differential effects across SEP groups.

The most important finding was that combining nudges with salient price strategies increased the proportion of healthy purchases in all three pricing arms. These results are in line with a previous study that found that price discounts and communication alone did not increase the sales of targeted products, while the combined approach statistically significantly increased the sales of promoted items [30]. Combining pricing and nudging strategies may have a multiplicative effect because multiple intervention strategies target multiple levels of influence. Indeed, lessons learned from tobacco and alcohol control strategies indicate that it is necessary to address not only the affordability, but also availability and acceptability of such products [31]. Therefore, combining pricing strategies (affordability) with strategies that encourage healthy foods through nudging (through availability and perhaps acceptability) may be more promising for increasing healthy food purchasing behavior than nudging or pricing strategies alone. Additionally, people may have different reasons to change and subsequently maintain their behavior. It is therefore important that interventions target as many pathways as possible. The relatively small effects of these independent pathways could then add up to an effect size relevant at a population level. Nudging and pricing policy measures may be seen as interfering with personal choice and the freedom to consume. However, they can also be seen as interventions enabling the freedom and right to health and a healthy environment by rebalancing and prioritizing the right to health over the right to consume [32].

Another finding in this study is that salient pricing strategies are more effective than non-salient price changes in increasing the proportion of healthy purchases. A previous experimental study among women found that salient subsidies alone and salient subsidies combined with taxes both increased healthy food purchases [33]. These results suggest that the absolute price of food is not the only driver influencing food purchasing behavior and that it may be important to communicate price changes to consumers. Salient price increases/taxes and discounts/subsidies may more accurately reflect real-life situations as well, as price changes are often communicated within mass media, governmental agent announcements and/or labels on supermarket shelves. What method of communication about price changes is most effective in reducing unhealthy purchases and increasing healthy purchases remains to be investigated. Other than communicating price changes, the results also suggest that it is important to combine price increases and discounts. Subsidies alone may lead to an increase in overall calorie consumption [34] and taxes may be more accepted by the public when the tax revenue is earmarked to subsidize healthy foods [35]. Therefore, combining subsidies with taxes may be more effective than implementing just one of the two strategies. Alternatively, minimum pricing on confectionary foods may be implemented. This strategy is already applied to alcoholic beverages, where the minimum unit pricing on alcohol sets a floor price for a unit of alcohol, meaning that it cannot be sold for lower than the given floor price [36].

Non-statistically significant effects of nudging and non-salient pricing strategies have previously been reported. For example, a systematic review found that only 5 out of 11 studies using salience-type nudges were effective [37]. Combining different types of nudges (e.g., salience and priming nudges) may be more effective in influencing food purchasing behavior [37], especially in environments full of food cues such as supermarkets. Also, previous experimental studies have found mixed results regarding the effect of non-salient taxes and/or subsidies on increasing healthy beverage and food purchasing behavior [16, 38]. A possible explanation for the non-statistically significant effects of non-salient pricing strategies may be that participants did not have an accurate reference price due to shopping in a new supermarket environment. Even though the virtual supermarket was rated positively by participants [17], it may have been more difficult to read price tags on a computer screen compared to real-life.

Nudging and pricing strategies would be considerably less appropriate if people who already benefit from personal resources such as higher education or higher food budgets were the ones who responded best to the intervention, thereby increasing socioeconomic inequalities in diet. In this study we found limited differences across low and high SEP regarding the effect of nudging and/or pricing strategies on food purchases. An example of a difference between the two groups was the stronger effect of salient price discounts (with and without nudges) on healthy food purchases for participants with a low income compared to a high income, but not for participants with a low compared to high educational level. While, as far as we are aware, no comparable studies have investigated moderation by income or educational level in the association between salience nudges and food purchases, studies investigating the moderating role of SEP proxies in the association between pricing strategies and food purchasing behavior also found no differential effects by educational level and income [15, 16]. It may be possible that low and high SEP populations indeed do not respond differently to pricing strategies. However, our low SEP group was still relatively heterogeneous in education level (including both low and medium educated participants [17]). Therefore, caution is needed with generalizing these conclusions to those with the lowest SEP.

Although the nudging and pricing strategy effects may be relatively small on an individual level, they can translate into relevant changes at a population level. When combining salient price increases with discounts, healthy household purchases increased by a maximum of 1646 g (in the price salience and nudging condition) and unhealthy household purchases decreased by a maximum of 1681 g (in the price salience condition). This translates to approximately an 80 g increase in healthy foods and an 80 g decrease in unhealthy foods per person per day (i.e., by dividing the overall change in healthy/unhealthy purchases per day by the mean number of adults and children within a household). Given that the Dutch population consumes on average 268 g of healthy products per day [26], an 80 g increase in healthy purchases seems relevant from a public health perspective (assuming that purchases are comparable to consumption [39]).

However, further research into the single and combined effects of nudging and pricing strategies (especially price increases) in real-world settings and on long-term disease outcomes is needed. Therefore, we are currently planning on conducting an intervention within the Supreme Nudge project which aims to investigate the effect of nudging and pricing strategies on cardiometabolic disease risk in adults with a low SEP [25]. The pricing strategies of this real-life study will include a price reduction of 25% on healthy foods and a price difference of 25% between unhealthy products and their healthier substitutes within the same food group (e.g., white bread and whole-grain bread) [25]. Next to investigating the effect of pricing and nudging strategies in real-world settings, further research should investigate possible differential effects of nudging and pricing strategies by factors influencing purchasing habits (e.g., single-member households, age, impulsiveness and price sensitivity).

### Strengths and limitations

The effectiveness of (salient) price increases have, as far as we are aware, not yet been studied within randomized controlled trials in real supermarket environments. One possible reason for the lack of real-life experimental studies may be the risk that the proposed pricing strategies negatively influence profits. Indeed, we found that participants spent considerably and significantly less when receiving a 25% discount. Therefore, a strong merit of this study is the use of the virtual supermarket tool that closely simulates real-life experiences, which allowed us to collect objective data, behavioral measures and control manipulations. Another strength of this study includes the between-within subject design where participants acted as their own control, resulting in a statistically powerful analysis as factors that may have caused variability between subjects were controlled for by the repeated measures design. Lastly, a relatively high completion rate of 76% was found where study completers did not significantly differ from study non-completers [17].

A limitation of the present study includes the limited generalizability of the study results. The experimental research design has a limited external validity as the results may not be directly translatable to the real world. Another limitation is that the food budget used in the study were declarative (i.e., participants did not actually purchase the items purchased in the SN VirtuMart). This may undermine the experimental results due to for example social desirability bias. However, a study conducted by Waterlander et al. validated a virtual supermarket as a measure to collect food purchasing data in a supermarket setting by demonstrating that shopping patterns in a virtual supermarket resemble those in real life [19]. Also, 78% of participants indicated that they feel that their virtual supermarket purchases simulated real life purchases [17]. The limited generalizability of the results may also come from the relatively young and high SEP population included in the study, which does not reflect the average Dutch population [17]. The heterogeneity of the low SEP group in our study limits the opportunity to study the effects of nudging and pricing strategies in the lowest SEP group. Therefore, the results in this study may have underestimated SEP differences. Lastly, this study attempted to investigate the effect of nudging on purchasing behavior while only one type of nudge was implemented in the SN VirtuMart, which may have led to the limited evidence for nudges.

## Conclusion

This study demonstrated that salient price increases and discounts combined with nudges increase healthy food purchasing behavior, more so than each of the independent strategies. Moreover, nudging and/or pricing strategies do not seem to widen SEP inequalities. Further research is needed to investigate the single and combined effect of nudging and (salient) pricing strategies (especially taxes on unhealthy foods) in real-world settings.

## Supplementary information

**Additional file 1.**

## Data Availability

Requests for de-identified individual participant data or study documents will be considered where the proposed use aligns with public positive purposes, does not conflict with other requests or planned use by the steering committee, and the requestor is willing to sign a data access agreement. Contact is through the corresponding author. Consent for data sharing was not obtained but the presented data are anonymized and the risk of identification is low. The original protocol is available from the corresponding author on request.

## Abbreviations

3D: Three-dimensional
SNVirtuMart: Supreme Nudge Virtual Supermarket
SEP: Socio-economic Position

## References

[1] Popkin BM, Adair LS, Ng SW (2012). Global nutrition transition and the pandemic of obesity in developing countries. Nutr Rev.

[2] Duffey KJ, Popkin BM (2011). Energy density, portion size, and eating occasions: contributions to increased energy intake in the United States, 1977–2006. PLoS Med.

[3] Willett WC, Koplan JP, Nugent R, Dusenbury C, Puska P, Gaziano TA. Prevention of chronic disease by means of diet and lifestyle changes. In: Disease Control Priorities in Developing Countries 2nd edition. New york: The International Bank for Reconstruction and Development/The World Bank; 2006. 21250366

[4] Backholer K, Sacks G, Cameron AJ. Food and beverage Price promotions: an untapped policy target for improving population diets and health. Curr Nutr Rep. 2019;8:250–5. 10.1007/s13668-019-00287-z 31300982

[5] Hartmann-Boyce J, Bianchi F, Piernas C, Riches S, Frie K, Nourse R, Jebbs S (2019). Grocery store interventions to change food purchasing behaviors: a systematic review of randomized controlled trials. Am J Clin Nutr.

[6] Scarborough P, Adhikari V, Harrington RA, Elhussein A, Briggs A, Rayner M, Adams J, Cummins S, Penney T, White M (2020). Impact of the announcement and implementation of the UK soft drinks industry levy on sugar content, price, product size and number of available soft drinks in the UK, 2015-19: a controlled interrupted time series analysis. PLoS Med.

[7] Afshin A, Penalvo JL, Del Gobbo L, Silva J, Michaelson M, O'Flaherty M, Capewell S, Spiegelman D, Danaei G, Mozaffarian D (2017). The prospective impact of food pricing on improving dietary consumption: a systematic review and meta-analysis. PLoS One.

[8] Anderson E, Simester D (2003). Mind your pricing cues. Harv Bus Rev.

[9] Cornelsen L, Mazzocchi M, Smith RD (2019). Fat tax or thin subsidy? How price increases and decreases affect the energy and nutrient content of food and beverage purchases in Great Britain. Soc Sci Med.

[10] Thaler RH, Sunstein CR. Nudge: improving decisions about health, wealth, and happiness. London: Penguin Putnam Inc; 2008.

[11] Evers C, Marchiori DR, Junghans AF, Cremers J, De Ridder DTD (2018). Citizen approval of nudging interventions promoting healthy eating: the role of intrusiveness and trustworthiness. BMC Public Health.

[12] Arno A, Thomas S (2016). The efficacy of nudge theory strategies in influencing adult dietary behaviour: a systematic review and meta-analysis. BMC Public Health.

[13] Vecchio R, Cavallo C (2019). Increasing healthy food choices through nudges: a systematic review. Food Qual Prefer.

[14] Darmon N, Lacroix A, Muller L, Ruffieux B (2016). Food Price policies may improve diet but increase socioeconomic inequalities in nutrition. World Rev Nutr Diet.

[15] Blakely T, Ni Mhurchu C, Jiang Y, Matoe L, Funaki-Tahifote M, Eyles HC, Foster RH, McKenzie S, Rodgers A (2011). Do effects of price discounts and nutrition education on food purchases vary by ethnicity, income and education? Results from a randomised, controlled trial. J Epidemiol Community Health.

[16] Waterlander WE, Steenhuis IH, de Boer MR, Schuit AJ, Seidell JC (2012). The effects of a 25% discount on fruits and vegetables: results of a randomized trial in a three-dimensional web-based supermarket. Int J Behav Nutr Phys Act.

[17] Hoenink JC, Mackenbach JD, Van der Laan LN, Lakerveld J, Waterlander W, Beulens JWJ: Recruitment of participants for a virtual supermarket study. Submitted to JMIR Formative Research. 2020. 10.2196/preprints.19234. 10.2196/19234 PMC7902190 33560230

[18] Mizdrak A, Waterlander WE, Rayner M, Scarborough P (2017). Using a UK virtual supermarket to examine purchasing behavior across different income groups in the United Kingdom: development and feasibility study. J Med Internet Res.

[19] Waterlander WE, Jiang Y, Steenhuis IH, Ni Mhurchu C (2015). Using a 3D virtual supermarket to measure food purchase behavior: a validation study. J Med Internet Res.

[20] Herpen E, van den Broek E, van Trijp HCM, Yu T (2016). Can a virtual supermarket bring realism into the lab? Comparing shopping behavior using virtual and pirctorial store representations to behavior in a physical store. Appetite.

[21] Lakerveld J, Mackenbach JD, de Boer F, Brandhorst B, Broerse JEW, de Bruijn GJ, Feunekes G, Gillebaart M, Harbers M, Hoenink J (2018). Improving cardiometabolic health through nudging dietary behaviours and physical activity in low SES adults: design of the supreme nudge project. BMC Public Health.

[22] Kromhout D, Spaaij CJ, de Goede J, Weggemans RM (2016). The 2015 Dutch food-based dietary guidelines. Eur J Clin Nutr.

[23] 70% supermarkt bestaat uit omstreden ‘ultra-processed foods’ (70% of supermarkets consist of ‘ultra-processed foods’). https://www.foodwatch.org/nl/persberichten/2017/70-supermarkt-bestaat-uit-omstreden-ultra-processed-foods/. Accessed 1 June 2020.

[24] Mytton OT, Clarke D, Rayner M (2012). Taxing unhealthy food and drinks to improve health. Bmj.

[25] Stuber JM, Mackenbach JD, de Boer FE, de Bruijn G-J, Gillebaart M, Harbers MC, Hoenink JC, Klein MC, Middel CN, van der Schouw YT (2020). Reducing cardiometabolic risk in adults with a low socioeconomic position: protocol of the supreme nudge parallel cluster-randomised controlled supermarket trial. Nutr J.

[26] Van Rossum CTM, Buurma Rethans EJM, Vennemann FBC, Beukers M, Brants HAM, de Boer EJ, Ocké MC. The diet of the Dutch : results of the first two years of the Dutch National Food Consumption Survey 2012–2016. Bilthoven: RIVM; 2016.

[27] Hagenaars LL, Jeurissen PPT, Klazinga NS (2017). The taxation of unhealthy energy-dense foods (EDFs) and sugar-sweetened beverages (SSBs): an overview of patterns observed in the policy content and policy context of 13 case studies. Health Policy.

[28] Inkomen van personen (Personal income). https://longreads.cbs.nl/welvaartinnederland-2019/inkomen-van-personen/.

[29] Lennernas M, Fjellstrom C, Becker W, Giachetti I, Schmitt A (1997). Remaut de winter a, Kearney M: influences on food choice perceived to be important by nationally-representative samples of adults in the European Union. Eur J Clin Nutr.

[30] Budd N, Jeffries JK, Jones-Smith J, Kharmats A, McDermott AY, Gittelsohn J (2017). Store-directed price promotions and communications strategies improve healthier food supply and demand: impact results from a randomized controlled, Baltimore City store-intervention trial. Public Health Nutr.

[31] Capewell S, Lloyd-Williams F (2017). Promotion of healthy food and beverage purchases: are subsidies and consumer education sufficient?. Lancet Public Health.

[32] Purdie A, Buse K, Hawkes S (2019). In Syntax and the “sin tax”: the power of narratives for health.

[33] Muller L, Lacroix A, Lusk JL (2017). Distributional impacts of fat taxes and thin subsidies. Econ J.

[34] Thow AM, Downs S, Jan S (2014). A systematic review of the effectiveness of food taxes and subsidies to improve diets: understanding the recent evidence. Nutr Rev.

[35] Tamir O, Cohen-Yogev T, Furman-Assaf S, Endevelt R (2018). Taxation of sugar sweetened beverages and unhealthy foods: a qualitative study of key opinion leaders’ views. Isr J Health Policy Res.

[36] Sharma A, Vandenberg B, Hollingsworth B (2014). Minimum pricing of alcohol versus volumetric taxation: which policy will reduce heavy consumption without adversely affecting light and moderate consumers?. PLoS One.

[37] Wilson AL, Buckely E, Buckley JD, Bogomolova S (2016). Nudging healthier food and beverage choices through salience and priming. Evidence from a systematic review. Food Qual Prefer.

[38] Giesen JC, Havermans RC, Nederkoorn C, Jansen A (2012). Impulsivity in the supermarket. Responses to calorie taxes and subsidies in healthy weight undergraduates. Appetite.

[39] Appelhans BM, French SA, Tangney CC, Powell LM, Wang Y (2017). To what extent do food purchases reflect shoppers’ diet quality and nutrient intake?. Int J Behav Nutr Phys Act.

