# Supplementary material for: The effects of nudging and pricing on healthy food purchasing behavior in a virtual supermarket setting: a randomized experiment
Source: Int J Behav Nutr Phys Act. 2020 Aug 3;17:98. doi: 10.1186/s12966-020-01005-7 (PMC7398383; doi:10.1186/s12966-020-01005-7)
Supplement: Supplementary file 1 — Additional file 1. [file 12966_2020_1005_MOESM1_ESM.docx]

# **Additional files**

## The virtual supermarket program

The Supreme Nudge VirtuMart (SN VirtuMart) described in this study was developed by a member of the study group (NvdL). The SN VirtuMart was adapted from an existing virtual supermarket (1). NvdL changed the layout of the virtual supermarket to replicate an average Dutch Coop supermarket (i.e., a supermarket chain in the Netherlands, partnering in the larger Supreme Nudge project). The virtual version was designed such that it reflects a medium-sized supermarket where people can conduct their weekly shopping. The gaming development platform UNITY was used to construct the 3D computer-based virtual supermarket (1). 3D models of food and beverages were created in Blender and were designed to replicate real products (e.g., branding, size, shape, color, and style of packaging). The nutrition information of products was not displayed within the SN VirtuMart: the front of the product was also used for the back of the product. In order to simulate real-life supermarket shopping experiences, common marketing, branding and promotion techniques as well as sounds and background noise were used. The SN VirtuMart could be downloaded using a zip file and unpacking this to install and open the SN VirtuMart program on either Windows or Apple computers.

## Functionalities and user controls

Functionalities included the ability to move forward, turn left and right, look around, bend, view a product and the price up close, view the physical shopping basket and view a list of products within the shopping basket. Participants could use their arrow keys to turn left and right, go forward and to bend. Additionally, the mouse could be used to change the camera’s orientation (to look around). Participants could view the functionalities at all times by pressing escape. Participants could directly select products by left-clicking on the product and put them in their basket or they could right-click on the product to view a close-up of a product and then select the product to go into the basket. Participants were able to leave the virtual supermarket environment by walking to the cash register or pressing escape and choosing the option to leave the supermarket.

## Nudges within the SN VirtuMart

We implemented salience nudges to stimulate the purchases of healthier products and the substitution of unhealthier products for healthier ones. The salience nudges included bright orange frames around healthy low fat dairy products, a frame around the door of the frozen fruits and vegetables and orange arrows pointing from unhealthy to healthier high fiber variants (Figure 1).

## Prices and budget within the SN VirtuMart

Food prices, food labels and food placing could be adapted in Unity via Excel or a text editor with the aim to create different research conditions. Participants’ shopping budgets were based on self-reported real-life shopping budgets and implemented in the SN VirtuMart. Participants needed to spend at least 50% of their allocated budget in order to prevent participants from purchasing just a few items and quit the experiment. Participants could also overspend to a maximum of 125% to allow for overspending in the taxing arms (2). Login codes were used to assign participants to certain conditions and budgets. Each week, during five consecutive weeks, participants received a new log in code. The log in codes were connected to a specific virtual shopping budget and a specific condition (e.g. control, nudging or pricing condition) within the virtual supermarket.

## Data collected in the SN VirtuMart

The virtual supermarket application stored information on time spent in the supermarket, participants’ walking routes through the supermarket, what products were looked at up-close, what products were placed into the shopping basket, what products were ultimately purchased and the total amount of money spent during a shop. Data was stored on both the participants’ computer as well as on the university server. Data was stored and sent to the server after participants clicked on the ‘leave supermarket’ button.

## Selection of food and beverage products

The SN VirtuMart included 1179 unique name-brand and budget-brand products categorized into 12 large food groups. Nonfood items and alcoholic beverages were excluded from the virtual supermarket. The SN VirtuMart did not include all food products that are normally present in a supermarket because it is not feasible to model all these products. Within each food category we selected top-selling products from an average Coop supermarket to be included in the stock of the virtual supermarket. The quantity and variety of products was such that participants with a variety of household sizes and budgets were able to do their weekly shopping in the virtual supermarket. Usual prices (i.e., excluding offers) for the selected products were collected from the Coop supermarket website in the summer of 2018.

***
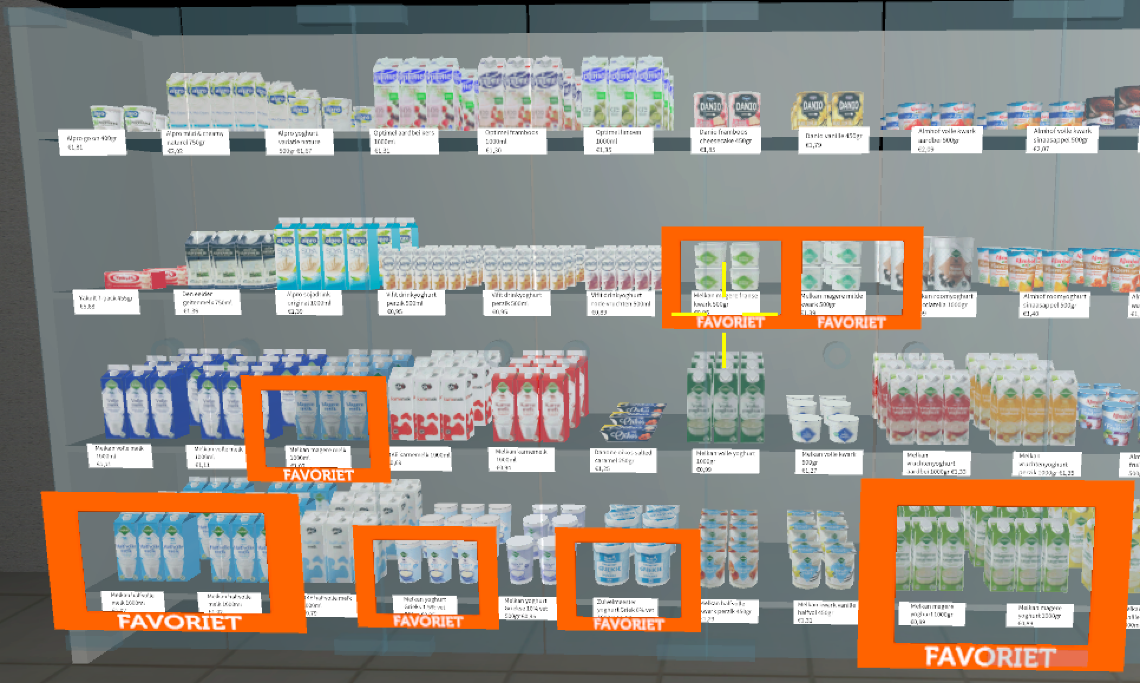

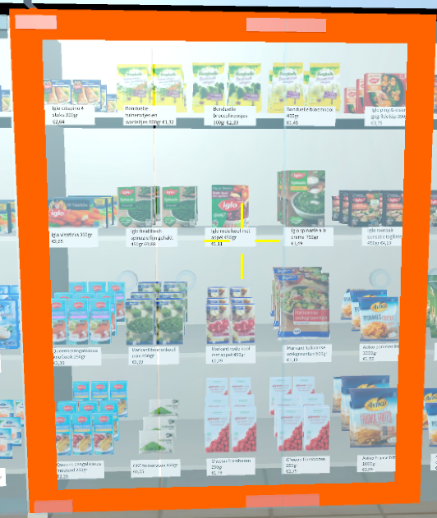

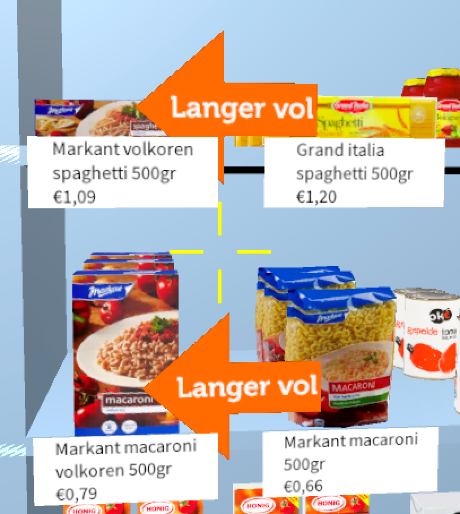
***

Figure 1. Three types of salience nudges used within the SN VirtuMart. Arrow pointing from a less healthy grain product to a healthy whole grain product (left), orange frame around the entire frozen fruit and vegetables door (middle) and small orange frames around low fat dairy products (right).

# References

1. Van der Laan LN, Papies EK, Ly A, Smeets PA. How health goal priming promotes healthy food choice: a virtual reality fMRI study. Submitted. 2019.

2. Waterlander WE, Jiang Y, Nghiem N, Eyles H, Wilson N, Cleghorn C, et al. The effect of food price changes on consumer purchases: a randomised experiment. Lancet Public Health. 2019;4(8):e394-e405

**Additional Table 1. Descriptive statistics of the outcome variables for the control condition**

| **Outcome variables** | **25% increase arm**  **(N= 119)** | **25% discount arm**  **(N= 129)** | **25% increase and discount arm**  **(N= 120)** | **Total sample**  **(N = 368)** |
| --- | --- | --- | --- | --- |
| Mean percentage of purchased healthy products in grams | 47.8% | 45.4% | 45.7% | 46.3% |
| Mean purchased healthy products in grams (SD) | 13591.5  (6441.1) | 13165.3  (6615.7) | 14559.4  (7296.7) | 13757.7  (6797.8) |
| Mean purchased unhealthy products in grams (SD) | 16014.1  (9483.6) | 16795.0  (10287.9) | 17839.9  (9415.1) | 16883.2  (9753.1) |
| Mean amount spent on total purchases in euros (SD) | 96.8  (42.6) | 98.3  (45.3) | 105.6  (45.0) | 100.2  (44.4) |

Abbreviations: SD; Standard Deviation

**Additional Table 2. Effects of nudging and several pricing strategies on healthy and unhealthy purchases stratified for arm and educational level**

| Conditions | 25% increase arm | | | | |  | 25% discount arm | | | |  | 25% increase and discount arm | | | |  |
| --- | --- | --- | --- | --- | --- | --- | --- | --- | --- | --- | --- | --- | --- | --- | --- | --- |
|  | **Low educational level**  **(n=65)** | | **High educational level**  **(n=63)** | | |  | **Low educational level**  **(n= 78)** | | **High educational level**  **(n=58)** | |  | **Low educational level**  **(n= 76)** | | **High educational level**  **(n=60)** | |  |
|  | **B** | **95%CI** | **B** | | **95%CI** | **lr-test** | **B** | **95%CI** | **B** | **95%CI** | **lr-test** | **B** | **95%CI** | **B** | **95%CI** | **lr-test** |
|  |  |  | | Healthy purchases in grams | | | | | | | | | | | |  |
| Control | Ref. | | Ref. | | | 0.1 | Ref. | | Ref. | | 0.3 | Ref. | | Ref. | | 0.2 |
| Nudging | 338.0 | -729.4; 1405.4 | **-1155.5** | | **-2218.7;**  **-92.3** |  | -954.1 | -1975.1; 66.8 | 557.1 | -585.8; 1700.1 |  | -818.1 | -1841.2; 204.9 | 1208.7 | -100.8; 2518.2 |  |
| Pricing | -383.7 | -1444.5; 677.0 | -1004.0 | | -2062.2; 54.2 |  | -480.3 | -1491.8; 531.2 | 725.8 | -432.3; 1883.9 |  | -474.8 | -1499.5; 549.8 | 333.3 | -994.3; 1660.9 |  |
| Price salience | 205.8 | -857.7; 1269.2 | -249.1 | | -1313.0; 814.7 |  | 444.1 | -572.4; 1460.6 | **1792.4** | **649.0; 2935.9** |  | 521.1 | -513.9; 1556.1 | **1683.4** | **345.9; 3020.8** |  |
| Price salience and nudging | 877.2 | -174.1; 1928.5 | -994.5 | | -2059.3; 70.3 |  | 63.6 | -947.1; 1074.3 | 914.6 | -221.9; 2051.1 |  | **1362.8** | **264.5; 2461.0** | **2063.1** | **657.2; 3469.1** |  |
|  |  |  | | Unhealthy purchases in grams | | | | | | | | | | | |  |
| Control | Ref. | | Ref. | | | 0.0 | Ref. | | Ref. | | 0.3 | Ref. | | Ref. | | 0.7 |
| Nudging | **-1788.1** | **-3203.4;**  **-372.8** | 632.0 | | -502.5; 1,928.5 |  | -952.2 | -2484.6; 580.3 | -508.8 | -1792.3; 774.7 |  | -1202.9 | -2630.2; 224.4 | -541.9 | -1752.9; 669.2 |  |
| Pricing | **-2626.3** | **-4032.1;**  **-1220.5** | 205.1 | | -949.7; 1,466.8 |  | -1130.0 | -2647.9; 387.9 | 226.5 | -1074.1; 1527.1 |  | -333.6 | -1763.1; 1095.8 | -966.5 | -2194.8; 261.9 |  |
| Price salience | -1271.7 | -2681.0; 137.5 | -766.2 | | -1,928.9; 500.2 |  | 81.9 | -1443.8; 1607.6 | 76.3 | -1207.8; 1360.5 |  | **-1572.7** | **-3016.6;**  **-128.7** | **-1817.1** | **-3053.9;**  **-580.3** |  |
| Price salience and nudging | **-2441.5** | **-3835.8;**  **-1047.2** | -1223.9 | | -2,386.7; 44.8 |  | **-1815.8** | **-3332.6;**  **-299.1** | 54.6 | -1221.8; 1331.0 |  | -1079.3 | -2611.4; 452.8 | -650.5 | -1950.2; 649.1 |  |

Bold values are statistically significant

Abbreviations: B; beta regression coefficient, CI; confidence interval, lr-test; p-value likelihood-ratio test

**Additional Table 3. Effects of nudging and several pricing strategies on healthy and unhealthy purchases stratified for arm and income level**

| Conditions | 25% increase arm | | | | |  | 25% discount arm | | | |  | 25% increase and discount arm | | | |  |
| --- | --- | --- | --- | --- | --- | --- | --- | --- | --- | --- | --- | --- | --- | --- | --- | --- |
|  | **Low income (n=75)** | | **High income (n=52)** | | |  | **Low income**  **(n=99)** | | **High income (n=35)** | |  | **Low income (n=92)** | | **High income (n=43)** | |  |
|  | **B** | **95%CI** | **B** | | **95%CI** | **lr-test** | **B** | **95%CI** | **B** | **95%CI** | **lr-test** | **B** | **95%CI** | **B** | **95%CI** | **lr-test** |
|  |  |  | | Healthy purchases in grams | | | | | | | | | | | |  |
| Control | Ref. | | Ref. | | | 0.7 | Ref. | | Ref. | | 0.3 | Ref. | | Ref. | | 0.3 |
| Nudging | -97.2 | -1118.8; 924.3 | -837.7 | | -1983.8; 308.4 |  | -422.9 | -1341.6; 495.8 | 102.4 | -1273.7; 1478.5 |  | -41.7 | -986.1; 902.7 | 140.1 | -1417.7; 1697.9 |  |
| Pricing | -448.9 | -1475.9; 578.1 | -1019.9 | | -2143.1; 103.4 |  | -368.8 | -1284.7; 547.2 | 1281.3 | -108.5; 2671.2 |  | -101.0 | -1051.0; 849.0 | -301.2 | -1873.4; 1271.0 |  |
| Price salience | 422.9 | -600.7; 1446.4 | -573.6 | | -1712.1; 564.8 |  | **1058.0** | **141.5; 1974.4** | 879.0 | -494.8; 2252.8 |  | 455.6 | -503.2; 1414.4 | **2003.9** | **418.9; 3588.9** |  |
| Price salience and nudging | 308.6 | -706.0; 1323.2 | -587.1 | | -1724.1; 550.0 |  | 333.5 | -582.2; 1249.2 | 784.0 | -576.8; 2144.7 |  | **1374.5** | **342.6; 2406.5** | **2013.2** | **406.1; 3620.3** |  |
|  |  |  | | Unhealthy purchases in grams | | | | | | | | | | | |  |
| Control | Ref. | | Ref. | | | 0.5 | Ref. | | Ref. | | 0.1 | Ref. | | Ref. | | 0.6 |
| Nudging | -189.5 | -1378.0; 998.9 | -1014.0 | | -2569.3; 541.0 |  | **-1295.0** | **-2559.7;**  **-30.3** | 704.7 | -1004.3; 2413.8 |  | **-1251.8** | **-2354.1;**  **-149.5** | -246.1 | -2102.4; 1610.2 |  |
| Pricing | -861.4 | -2055.3; 332.4 | **-1710.2** | | **-3234.6;**  **-185.9** |  | -1220.9 | -2481.3;  39.6 | 1194.4 | -531.7; 2920.6 |  | -756.7 | -1865.7; 352.3 | -189.2 | -2062.8; 1684.4 |  |
| Price salience | -246.5 | -1436.1; 943.1 | **-1998.4** | | **-3543.1;**  **-453.7** |  | -501.0 | -1762.2; 760.2 | **1759.0** | **52.9;**  **3465.1** |  | **-2024.9** | **-3143.9;**  **-905.9** | -1000.1 | -2888.9; 888.8 |  |
| Price salience and nudging | **-1463.2** | **-2643.2;**  **-283.2** | **-2217.3** | | **-3761.3;**  **-673.4** |  | **-1802.3** | **-3062.6.;**  **-542.0** | 1092.7 | -597.3; 2782.8 |  | **-1548.1** | **-2752.2;**  **-344.0** | 259.8 | -1656.7; 2176.3 |  |

Bold values are statistically significant

Abbreviations: B; beta regression coefficient, CI; confidence interval, lr-test; p-value likelihood-ratio test

**Additional Table 4. Effects of nudging and several pricing strategies on the primary outcome and secondary outcome measures for the total sample^1^ and stratified for pricing arms**

| **Conditions** | **25% increase arm (n=128)** | | | **25% discount arm (n=136)** | | **25% increase and discount arm (n=136)** | | **Total sample (n=400)** | |
| --- | --- | --- | --- | --- | --- | --- | --- | --- | --- |
|  | **B** | **95%CI** | **B** | | **95%CI** | **B** | **95%CI** | **B** | **95%CI** |
|  | Percentage of healthy purchases | | | | | | | | |
| Control | Ref. | | Ref. | | | Ref. | | Ref. | |
| Nudging | -0.3 | -2.0; 1.3 | 0.6 | | -1.2; 2.3 | 1.6 | -0.0; 3.3 | 0.6 | -0.4; 1.6 |
| Pricing | 0.6 | -1.1; 2.2 | 1.0 | | -0.7; 2.8 | 1.4 | -0.3; 3.1 | 1.0 | -0.0; 2.0 |
| Price salience | 1.2 | -0.4; 2.9 | **2.5** | | **0.7; 4.3** | **5.5** | **3.8; 7.2** | **3.1** | **2.1; 4.1** |
| Price salience and nudging | **2.7** | **1.1; 4.4** | **2.6** | | **0.8; 4.3** | **5.4** | **3.6; 7.2** | **3.5** | **2.5; 4.5** |
|  | Number of healthy purchases | | | | | | | | |
| Control | Ref. | | Ref. | | | Ref. | | NA | |
| Nudging | -0.4 | -1.6; 0.8 | -0.1 | | -1.4; 1.2 | 0.2 | -1.2; 1.6 | NA | NA |
| Pricing | -0.5 | -1.7; 0.7 | 0.6 | | -0.7; 1.9 | 0.2 | -1.2; 1.6 | NA | NA |
| Price salience | 0.0 | -1.2; 1.2 | **2.6** | | **1.3; 3.9** | **2.6** | **1.2; 4.0** | NA | NA |
| Price salience and nudging | -0.2 | -1.5; 1.0 | **1.6** | | **0.3; 2.9** | **3.2** | **1.7; 4.7** | NA | NA |
|  | Number of unhealthy purchases | | | | | | | | |
| Control | Ref. | | Ref. | | | Ref. | | NA | |
| Nudging | -0.7 | -2.6; 1.2 | -1.4 | | -3.6; 0.8 | -1.4 | -3.4; 0.6 | NA | NA |
| Pricing | **-2.0** | **-3.9; -0.2** | -1.5 | | -3.7; 0.7 | -1.8 | -3.8; 0.2 | NA | NA |
| Price salience | **-2.0** | **-3.9; -0.1** | 0.1 | | -2.2; 2.3 | **-3.8** | **-5.8; -1.8** | NA | NA |
| Price salience and nudging | **-3.8** | **-5.6; -1.9** | -2.2 | | -4.4; 0.0 | **-3.7** | **-5.8; -1.6** | NA | NA |

^1^ Only applicable for the primary outcome measure percentage of healthy purchases

^2^ Price salience condition significantly differs from pricing condition

Bold values are statistically significant

Abbreviations: B; beta regression coefficient, CI; confidence interval, NA; Not Applicable

**Additional Table 5. Effects of nudging and pricing strategies on the total amount spent on food purchases stratified for pricing arms**

| **Conditions** | **25% increase arm**  **(n=128)** | | **25% discount arm**  **(n=136)** | | **25% increase and discount arm (n=136)** | |
| --- | --- | --- | --- | --- | --- | --- |
|  | **B** | **95%CI** | **B** | **95%CI** | **B** | **95%CI** |
|  | Total amount spent on food purchases in € | | | | | |
| Control | Ref. | | Ref. | | Ref. | |
| Nudging | -3.6 | -8.1; 1.0 | -3.4 | -7.6; 0.8 | -1.7 | -5.9; 2.6 |
| Pricing | -0.0 | -4.6; 4.5 | **-7.2** | **-11.4; -3.0** | **-4.3** | **-8.5; -0.0** |
| Price salience | 0.4 | -4.1; 5.0 | -3.4 | -7.7; 0.8 | **-5.0** | **-9.3; -0.6** |
| Price salience and nudging | -2.5 | -7.1; 2.0 | **-9.2** | **-13.4; -5.0** | -4.5 | -9.0; 0.1 |

Bold values are statistically significant

Abbreviations: B; beta regression coefficient, CI; confidence interval

**Additional Table 6. Effects of nudging and pricing strategies for the percentage of healthy purchases stratified for arm and sex**

| Conditions | 25% increase arm | | | | |  | 25% discount arm | | | |  | 25% increase and discount arm | | | | | |  |
| --- | --- | --- | --- | --- | --- | --- | --- | --- | --- | --- | --- | --- | --- | --- | --- | --- | --- | --- |
|  | **Males (n=52)** | | **Females (n=76)** | | |  | **Males (n=56)** | | **Females (n=80)** | |  | **Males (n=47)** | | | **Females (n=89)** | | |  |
|  | **B** | **95%CI** | **B** | **95%CI** | | **lr-test** | **B** | **95%CI** | **B** | **95%CI** | **lr-test** | **B** | **95%CI** | | **B** | **95%CI** | **lr-test** | |
|  | Percentage of healthy purchases | | | | | | | | | | | | | | | | | |
| Control | Ref. | | Ref. | | | 0.1 | Ref. | | Ref. | | 0.2 | Ref. | | | Ref. | | | 0.8 |
| Nudging | -2.0 | -5.3; 1.3 | 0.7 | | -1.7; 3.1 |  | -2.0 | -5.1; 1.2 | 1.6 | -0.9; 4.1 |  | 1.5 | -1.4; 4.5 | | 1.9 | -0.5; 4.3 | |  |
| Pricing | -0.6 | -3.9; 2.7 | 0.5 | | -1.9; 2.9 |  | -1.8 | -4.9; 1.3 | 2.1 | -0.5; 4.6 |  | 0.4 | -2.5; 3.3 | | 0.8 | -1.6; 3.3 | |  |
| Price salience | 2.5 | -0.8; 5.8 | 0.6 | | -1.8; 3.0 |  | -0.7 | -3.9; 2.4 | **3.6** | **1.1; 6.2** |  | 2.9 | -0.1; 5.8 | | **5.3** | **2.8; 7.7** | |  |
| Price salience and nudging | **4.0** | **0.8; 7.3** | 2.3 | | -0.1; 4.7 |  | 1.2 | -1.9; 4.3 | **3.1** | **0.5; 5.6** |  | **3.6** | **0.5; 6.6** | | **4.3** | **1.7; 6.9** | |  |
|  | Healthy purchases in grams | | | | | | | | | | | | | | | | | |
| Control | Ref. | | Ref. | | | 0.4 | Ref. | | Ref. | | 0.2 | Ref. | | Ref. | | | | 0.5 |
| Nudging | -723.3 | -1783.4; 336.9 | -169.9 | | -1226.0; 886.2 |  | -551.2 | -1786.2; 683.7 | -121.0 | -1085.2; 843.1 |  | 637.8 | -646.5; 1922.0 | | -194.5 | -1229.9; 840.9 | |  |
| Pricing | -727.9 | -1784.7; 328.9 | -672.4 | | -1721.8; 376.9 |  | -120.6 | -1343.8; 1102.7 | 162.8 | -808.1; 1133.7 |  | -544.2 | -1822.6; 734.1 | | 72.8 | -975.5; 1121.1 | |  |
| Price salience | 178.4 | -885.3; 1242.1 | -167.7 | | -1218.9; 883.6 |  | 440.9 | -785.8; 1666.8 | **1426.9** | **461.9; 2391.9** |  | 762.5 | -526.4; 2051.5 | | **1156.2** | **98.2; 2214.2** | |  |
| Price salience and nudging | 514.8 | -534.2; 1563.9 | -458.0 | | -1510.2; 594.1 |  | 952.4 | -269.0; 2137.9 | 120.1 | -837.9; 1078.2 |  | **1902.0** | **559.0; 3244.9** | | **1548.0** | **422.4; 2673.6** | |  |
|  | Unhealthy purchases in grams | | | | | | | | | | | | | | | | | |
| Control | Ref. | | Ref. | | | 0.7 | Ref. | | Ref. | | 0.6 | Ref. | | Ref. | | | | 0.7 |
| Nudging | -278.6 | -1884.7; 1327.6 | -712.8 | | -1847.6; 422.0 |  | -286.0 | -2004.9; 1433.0 | -1063.4 | -2349.3; 222.5 |  | -325.9 | -2006.7; 1355.0 | | **-1176.6** | **-2343.8;**  **-9.3** | |  |
| Pricing | -791.0 | -2390.1; 808.2 | **-1474.0** | | **-2601.4;**  **-346.6** |  | 110.2 | -1592.2; 1812.7 | -963.2 | -2257.9; 331.5 |  | -197.3 | -1870.9; 1476.3 | | -814.0 | 1995.8; 367.8 | |  |
| Price salience | -1068.4 | -2678.2; 541.3 | -923.4 | | -2052.5; 205.6 |  | 815.0 | -891.4; 2521.4 | -367.8 | -1654.7; 919.1 |  | -1005.0 | -2692.5; 682.6 | | **-2010.2** | **-3202.7;**  **-817.6** | |  |
| Price salience and nudging | **-2121.2** | **-3708.5;**  **-534.0** | **-1568.1** | | **-2699.9;**  **-436.4** |  | 35.9 | -1664.1; 1735.9 | **-1650.3** | **-2927.8;**  **-372.8** |  | 93.0 | -1665.4; 1851.5 | | **-1376.3** | **-2645.1;**  **-107.4** | |  |

Bold values are statistically significant

Abbreviations: B; beta regression coefficient, CI; confidence interval, lr-test; P-value likelihood-ratio test

**Additional Table 7. Effect of the intervention period on the percentage of healthy purchases in the total sample**

|  | **Total sample (n=400)** | |
| --- | --- | --- |
|  | **B** | **95%CI** |
| Week 1 | Ref. | |
| Week 2 | 0.4 | -0.7; 2.6 |
| Week 3 | -0.4 | -1.6; 0.7 |
| Week 4 | 0.4 | -0.8; 1.6 |
| Week 5 | 0.9 | -0.3; 2.1 |

Abbreviations: B; beta regression coefficient, CI; confidence interval, Ref; Reference group

**Additional Table 8. Unadjusted and adjusted effects of nudging and several pricing strategies on the percentage of healthy purchases for the total sample**

| **Conditions** | **Unadjusted model** | | | **Adjusted model^1^** | |
| --- | --- | --- | --- | --- | --- |
|  | **B** | **95%CI** | **B** | | **95%CI** |
| Control | Ref. | | Ref. | | |
| Nudging | 0.5 | -0.6; 1.6 | 0.6 | | -0.6; 1.7 |
| Pricing | 0.4 | -0.7; 1.6 | 0.5 | | -0.6; 1.6 |
| Price salience | **2.6** | **1.4; 3.7** | **2.7** | | **1.5; 3.8** |
| Price salience and nudging | **3.1** | **1.9; 4.3** | **3.2** | | **2.0; 4.3** |

^1^ Adjusted for the intervention period

Bold values are statistically significant

Abbreviations: B; beta regression coefficient, CI; confidence interval, Ref; Reference group
